# RACK1 is indispensable for porcine reproductive and respiratory syndrome virus replication and NF-κB activation in Marc-145 cells

**DOI:** 10.1038/s41598-018-21460-4

**Published:** 2018-02-14

**Authors:** Junlong Bi, Qian Zhao, Lingyun Zhu, Xidan Li, Guishu Yang, Jianping Liu, Gefen Yin

**Affiliations:** 1 College of Veterinary Medicine, Jilin University, Changchun, 130062 Jilin province China; 2 College of Veterinary Medicine, Yunnan Agricultural University, Kunming, 650201 Yunnan province China; 3 Center for Animal Disease Control and Prevention, Chuxiong City, 675000 Yunnan province China; 4 Yunnan Province Veterinary Biological Products Development Center, Baoshan, 678000 Yunnan Province China; 5 Karolinska Institute, Integrated Cardio Metabolic Centre (ICMC), Stockholm, SE-14157 Sweden

## Abstract

Porcine reproductive and respiratory syndrome virus (PRRSV) causes porcine reproductive and respiratory syndrome (PRRS), which is currently insufficiently controlled. RACK1 (receptor of activated protein C kinase 1) was first identified as a receptor for protein kinase C, with increasing evidence showing that the functionally conserved RACK1 plays important roles in cancer development, NF-κB activation and various virus infections. However, the roles of RACK1 during PRRSV infection in Marc-145 cells have not been described yet. Here we demonstrated that infection of Marc-145 cells with the highly pathogenic PRRSV strain YN-1 from our lab led to activation of NF-κB and upregulation of RACK1 expression. The siRNA knockdown of RACK1 inhibited PRRSV replication in Marc-145 cells, abrogated NF-κB activation induced by PRRSV infection and reduced the viral titer. Furthermore, knockdown of RACK1 could inhibit an ongoing PRRSV infection. We found that RACK1 is highly conserved across different species based on the phylogenetic analysis of mRNA and deduced amino acid sequences. Taken together, RACK1 plays an indispensable role for PRRSV replication in Marc-145 cells and NF-κB activation. The results would advance our further understanding of the molecular mechanisms underlying PRRSV infection in swine and indicate RACK1 as a promising potential therapeutic target.

## Introduction

Porcine reproductive and respiratory syndrome (PRRS) is characterized by respiratory disorders in piglets and reproductive failure in sows^1^. The disease is a major cause of economic loss for the swine industry worldwide^2^. The causative agent, porcine reproductive and respiratory syndrome virus (PRRSV) is a member of the family Arteriviridae, genus Arterivirus^3^. The PRRSV genome is about 15.4 kb in length, which has 9 open reading frames encoding 7 structural proteins and 14 nonstructural proteins^4^. Among all of the encoded viral proteins, NSP9 is a putative RNA-dependent RNA polymerase and plays central roles in viral replication^5^. An earlier study, using the yeast two-hybrid (Y2H) system, showed that RACK1 (receptor of activated protein C kinase 1) might interact with the PRRSV protein NSP9^6^.

RACK1 was first identified as a receptor for protein kinase C^7^ and subsequently shown to act as a scaffold protein that plays important roles in multiple signaling pathways, including Akt^8^, Hedgehog^9^, Wnt/β–catenin^10^ and NF-κB activation^11^, and consequently in cancer development^12,13^. The sequence and function of RACK1 is highly conserved across evolutionarily distant species (from human to fly). It plays vital roles at different phases of various virus infections^14^. For example, depletion of the influenza A virus matrix protein M1-binding protein RACK1 impaired virus release^15^. Evidence from yeast two hybrid data and pull-down experiments showed that the Nef protein of HIV can bind to the carboxyl terminal of RACK1^16^. Tandem affinity purification and mass spectrometry revealed that RACK1 interacts with HBc, while overexpression of RACK1 counteracted the proapoptotic activity of HBc^17^. RACK1 is also an essential determinant for hepatitis C virus translation and infection^14^. RACK1 from the shrimp *Penaeus monodon* was identified as a specific cellular target protein for VP9, a nonstructural protein of white spot syndrome virus (WSSV)^18^. Walleye dermal sarcoma virus Orf B functions through RACK1 and protein kinase C^19^. Mumps virus V protein has the ability to interact strongly with RACK1^20^. In addition, RACK1 plays an anti-apoptotic role during infectious bursal disease virus (IBDV) infection via interaction with VDAC2 and VP5. It suggests that VP5 sequesters RACK1 and VDAC2 in the apoptosis-inducing process^21^. Furthermore, together with integrin beta, RACK1 is involved in the cell entry of Bombyx mori cypovirus^22^. Additionally, RACK1 was discovered as one of the 16 interacting cellular proteins by the yeast two-hybrid system during a screen for classic swine fever virus (CSFV) NS5A interactive proteins in the cDNA library of the swine umbilical vein endothelial cell (SUVEC)^23^. RACK1 was also screened with CSFV E2 as bait protein by yeast two-hybrid from porcine alveolar macrophages (PAM cells) expression library, which was further confirmed by co-transformation, GST pull-down and laser confocal assays^24^.

However, there are no studies that define the role of RACK1 during PRRSV infection. Here we analyzed the sequence similarity of RACK1 (at both mRNA and amino acid levels) across diverse species, and then investigated how PRRSV infection in Marc-145 cells affects the expression level of RACK1. We further show that siRNA knockdown of RACK1 in Marc-145 cells downregulates PRRSV replication, abrogates NF-κB activation induced by PRRSV infection and reduces the viral titer.

## Results

### Phylogenetic analysis and multiple sequence alignment

**Figure 1:**
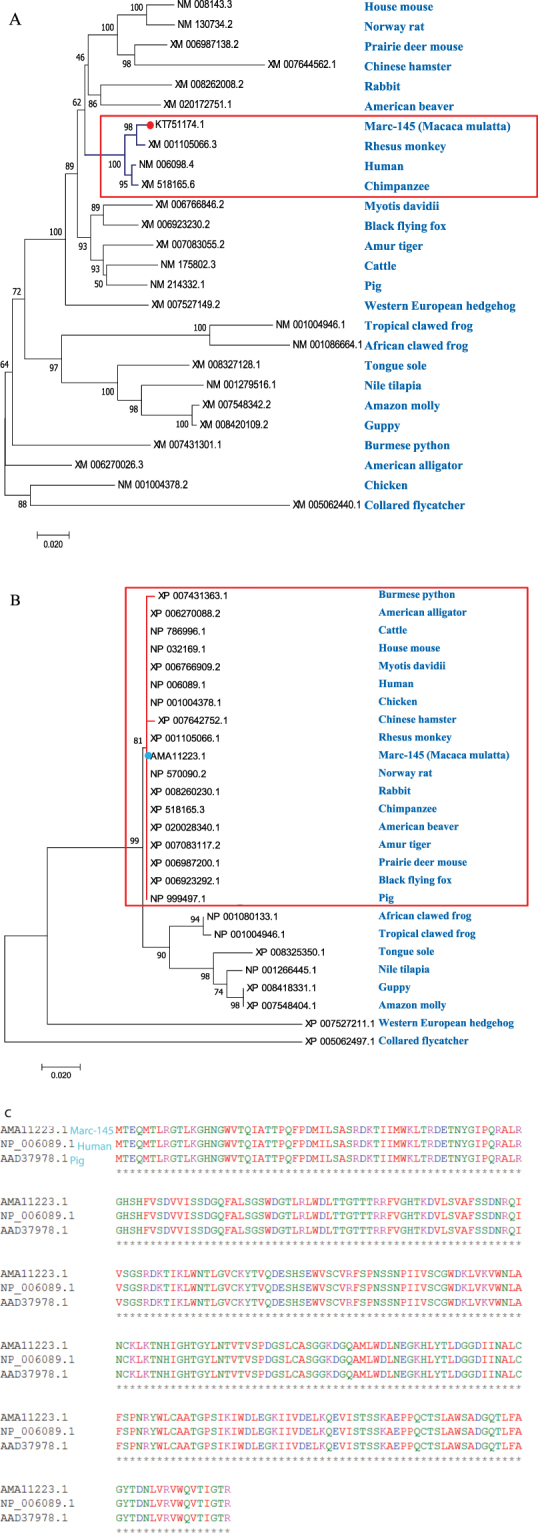
Phylogenetic analysis and multiple sequence alignment of RACK1 mRNA and amino acid sequences. (**A**) Phylogenetic analysis of RACK1 mRNA sequences from 26 species. (**B**) Phylogenetic analysis of RACK1 amino acid sequences from 26 species. (**C**) Multiple sequence alignment of RACK1 amino acid sequences from monkey, human and pig showed identical sequences. The sequence alignment and clustering was performed using the online tool Clustal Omega.

RACK1 mRNA sequence from Marc-145 cells (*Macaca mulatta*, a member of Rhesus monkey) is 954 nucleotides long (Genbank accession number KT751174.1) and encodes a protein sequence with a length of 317 amino acids. RACK1 orthologous mRNA sequences and amino acid sequences from 26 species, including the one sequenced at our lab from Marc-145 cells, were downloaded from The National Center for Biotechnology Information (NCBI) database. The phylogenetic analysis and multiple sequence alignment based on these data were performed using online tool Clustal Omega (https://www.ebi.ac.uk/Tools/msa/clustalo/) with the default parameters. The result showed that the RACK1 mRNA sequence of Marc-145 cells has a strong evolutionary connection with human and chimpanzee (indicated by the red square in Fig. 1A). Furthermore, the RACK1 amino acid sequence is also highly conserved between primates and additional species such as pig (highlighted in the red square in Fig. 1B,C).

### PRRSV infection upregulated cellular RACK1 expression

**Figure 2:**
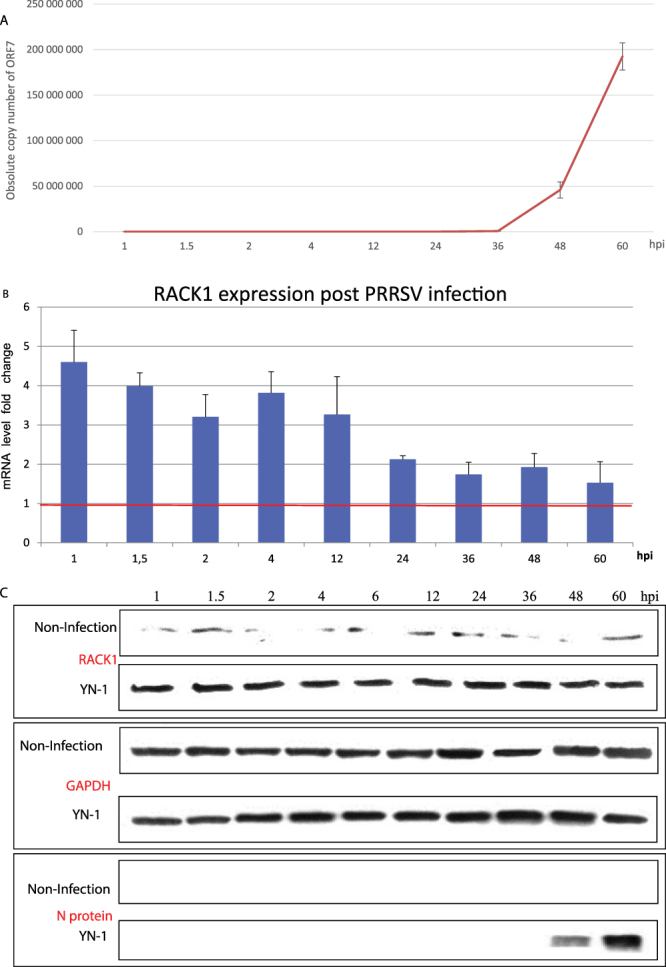
PRRSV infection in Marc-145 cells upregulated RACK1 expression on both mRNA and protein level. After challenge with PRRSV YN-1 strain (25 TCID_50_) in Marc-145 cells, total RNA was isolated at different time points for RT-qPCR analysis and the total protein was extracted for western blot analysis. (**A**) Absolute copy number of viral ORF7 mRNA over 60 hours’ infection time. The p value between the two datasets (1, 1.5, 2, 4, 12, 24, 36 hpi and 48, 60 hpi) is 0.0068, indicating a significant difference. (**B**) Relative mRNA expression level of RACK1. The ΔΔCt method for relative quantification of gene expression was used to determine viral ORF7 RNA levels. (**C**) Western blot analysis of RACK1 and viral N protein. After SDS-PAGE and protein transferring, the membranes were cut based on the molecular weights of the target proteins and the markers, put together along the same film for exposure with same exposure time for each sub-figure. The histograms and blots shown here are representative data from three independent experiments. GAPDH was used as internal control in both RT-qPCR and western blot analysis.

RACK1 expression is stimulus- and cell-type-specific. For instance, RACK1 is downregulated in EBV-infected monocytes^25^, while it is highly up-regulated through nerve growth factor (NGF) induced NF-κB activation^26^ or by TGF-β1 in mice^27^. Hence, in this study, we investigated the effect of PRRSV infection on the RACK1 expression. Marc-145 cells in 6-well plates were analysed at different time points post infection with 25 TCID_50_ of YN-1 strain or without virus challenge. RNA and proteins were extracted from the cells for virus copy determination, RACK1 mRNA and protein expression analysis. Copy number of PRRSV ORF7 mRNA significantly increased after 36 hours post infection (Fig. 2A) and viral N protein encoded by ORF7 was detected by western blot 48 hours post infection (Fig. 2C), indicating efficient virus replication in Marc-145 cells. During the whole experimental time period (from 1 hour to 60 hours), substantial increase of cellular RACK1 mRNA (Fig. 2B, uninduced levels highlighted by the red line) and protein (Fig. 2C) was observed, compared with the non-infection control. Even at the very beginning of PRRSV infection (such as 1–12 hours post virus inoculation), striking upregulation of RACK1 expression was recorded (Fig. 2C). However, we also observed an early increase of RACK1 mRNA production following PRRSV infection (Fig. 2B, 1 hpi), while RACK1 protein levels remain constant (Fig. 2C) throughout the period of infection. We reason that the mRNA expression data can not always correlate with the protein expression data in an exact manner.

### siRNA knockdown of RACK1 inhibited PRRSV replication and abrogated NF-κB activation induced by PRRSV infection

The transcription factor NF-κB plays a pivotal role in innate immunity in response to a variety of stimuli. When stimulated, the IκB proteins are phosphorylated by IκB kinases (IKKs) and degraded by proteasomes. Thus the IκB proteins allow the release and translocation of NF-κB into the nucleus to activate the transcription of genes involved in innate and adaptive immunity. The coordinated regulation of this pathway determines the proper host responses to extracellular signals^28–30^. Therefore, the authors investigated whether PRRSV infection in Marc-145 cells can activate NF-κB and whether RACK1 plays any role in the NF-κB activation.

**Figure 3:**
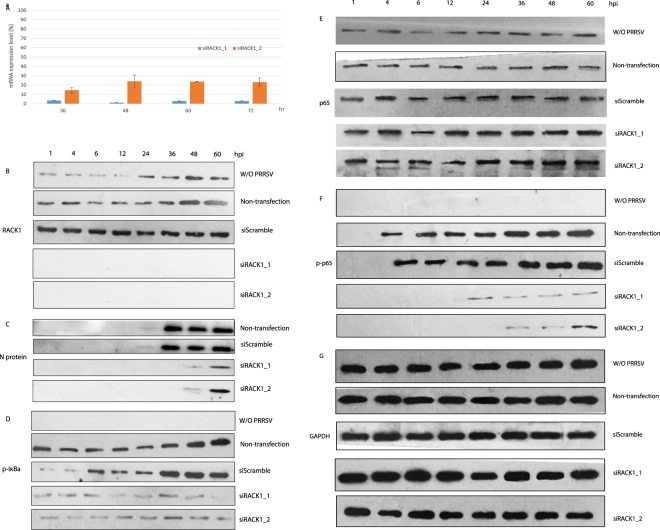
Knockdown of RACK1 abrogated PRRSV replication and NF-κB activation in Marc-145 cells. RACK1 mRNA expression level was measured by RT-qPCR at different time points post siRNA knockdown (**A**). The data was normalized to scramble siRNA treatment and the mRNA expression of internal control GAPDH. Forty-eight hours post siRNA knockdown, Marc-145 cells were inoculated with PRRSV YN-1 strain (25 TCID_50_). Total protein from various infection time points was analyzed by western blot for RACK1 (**B**), viral N protein (**C**), p-IκBα (**D**), p65 (**E**), p-p65 (**F**) and GAPDH (**G**). siRACK1_1 and siRACK1_2 are the two siRNA sequences (see Table 1) used in this study targeting different RACK1 mRNA sequences. After SDS-PAGE and protein transferring, the membranes were cut based on the molecular weights of the target proteins and the markers, put together along the same film for exposure with same exposure time for each sub-figure. The histograms and blots shown here are representative data from three independent experiments.

First, efficient knockdown was achieved using two siRNAs against RACK1, as demonstrated by RT-qPCR over 72 hours (Fig. 3A) and by western blot (Fig. 3B), when normalized to the internal GAPDH control and compared with the non-targeting siRNA transfection. Then two days post siRNA transfection, the Marc-145 cells cultured in 6-well plates were challenged by PRRSV (YN-1 strain, 25 TCID_50_). Total protein was collected at various time points post infection for western blot analysis. Compared with the non-infection treatment (w/o PRRSV), PRRSV infection constantly upregulated the RACK1 expression over a 60 hours duration (Fig. 3B, non-transfection and siScramble treatments), continuously induced the phosphorylation of IκBa and p65 (Fig. 3D,F), which became more significant with longer infection time. Our western blot data showing increased IκBα phosphorylation after PRRSV infection in Marc-145 cells (Fig. 3D) is in line with previous report in PAMs challenged with PRRSV or in CRL2843 cells transfected with N protein expression vector^31^, where phosphorylation of IκBa was observed already 30 minutes post infection or 24 hours post transfection. We further found that knockdown of RACK1 (Fig. 3B) resulted in clear inhibition of PRRSV replication (Fig. 3C) in a dose dependent manner (Supplementary Figure 1) and led to the abrogation of phosphorylation of IκBa and p65 (Fig. 3D,F), while no influence on the protein level of total p65 (Fig. 3E) was observed, suggesting that RACK1 downregulation blocked the NF-κB activation induced by PRRSV infection. We also ruled out the possibility that the anti-virus effects might come from the enhanced interferon alpha level resulted from the siRNA treatment (Supplementary Figure 2).

**Figure 4:**
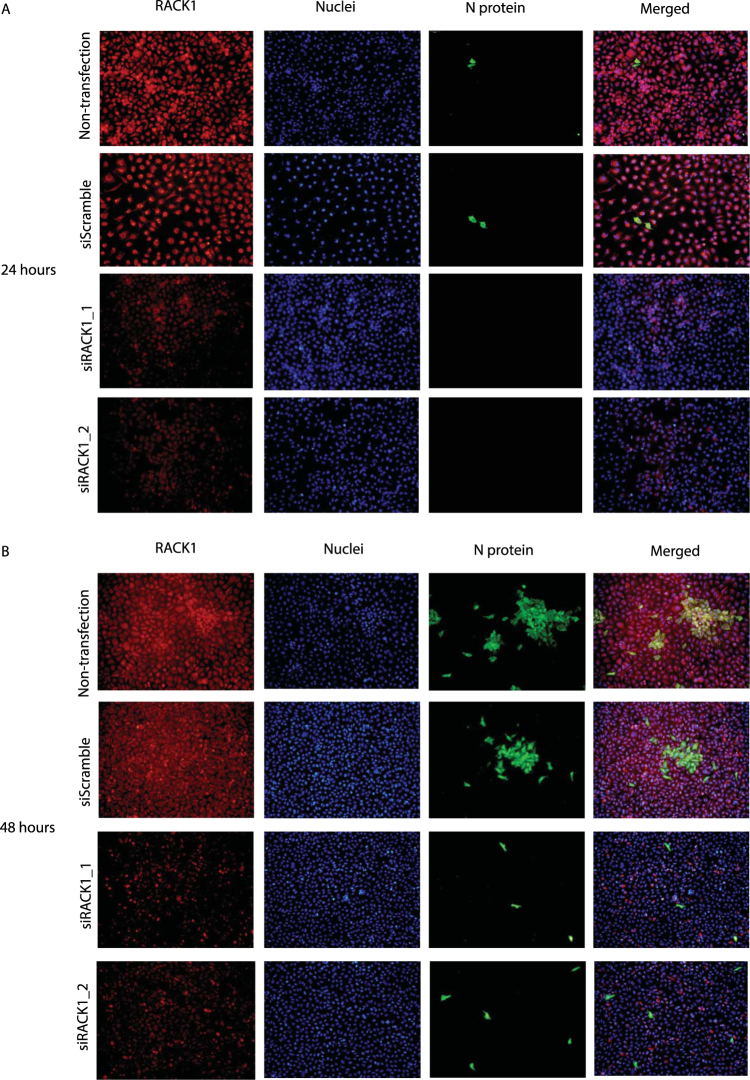
Indirect immunofluorescence detection of reduced PRRSV replication in Marc-145 cells transfected with siRNAs targeting RACK1. Transfection was performed 48 hours prior to infection with PRRSV YN-1 strain (25 TCID_50_). Cells were fixed 24 hours or 48 hours post infection. Anti-N monoclonal antibody (mAb) and Alexa Fluo488 conjugated secondary antibody were applied in indirect immunofluorescence staining to demonstrate the viral N protein level, with anti-RACK1 polyclonal antibody and Alexa Fluo546 conjugated secondary antibody to visualize the RACK1 protein level. Nuclei are shown in blue, RACK1 protein in red, and N protein in green. The images of the same treatment from the three channels were merged. These images are representative of three independent experiments.

In addition, immunofluorescent staining analysis was applied to further investigate the effect of RACK1 knockdown on PRRSV replication in Marc-145 cells. The cells were transfected with siRNAs, challenged with PRRSV 48 hours post transfection, fixed 24 hours or 48 hours post infection and immunofluorescently stained to visualize the nuclei (blue channel) and the expression level of RACK1 (red channel) and viral N protein (green channel). Reduction of RACK1 protein level occurred after siRNA knockdown with the two siRNA sequences (siRACK1_1 and siRACK1_2), compared with Non-transfection and siScramble groups (Fig. 4), which was more noticeable at 48 hours post transfection (Fig. 4B). Viral N protein was already detected 24 hours post infection in Non-transfection and siScramble groups (Fig. 4A), and increased to higher levels 48 hours post infection (Fig. 4B). RACK1 knockdown considerably (although not completely) inhibited the PRRSV infection, as deduced from a substantial reduction of the viral N protein level.

### Reduction of viral titer by knockdown of RACK1

**Figure 5:**
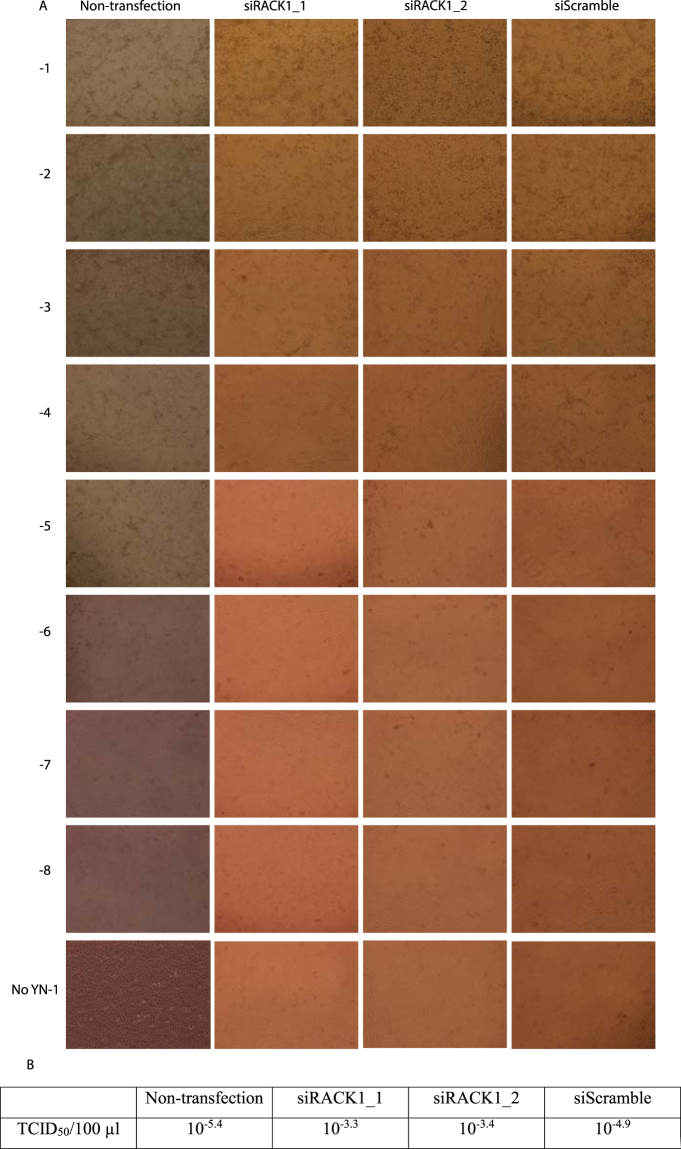
The siRNA knockdown of RACK1 protected Marc-145 cells from cytopathic effects and reduced the viral titer 40-fold compared with the non-targeting knockdown. Marc-145 cells (10^4^ cells per well) were seeded into 96-well plates the day of transfection. Forty eight hours post transfection, ten-fold serial dilution of PRRSV YN-1 strain was prepared in medium. One hundred µl/well of each dilution was added into 6 wells in total. CPE was recorded using the inverted microscope over a period of 3 days post transfection. The 50% tissue culture infected dose (TCID_50_) was determined by Reed–Muench method. Compared with the siScramble control group, transfection with siRACK1 significantly protected Marc-145 cells from cytopathic effects (**A**) and reduced the viral titer by nearly 40 times. These data is representative for three independent experiments.

Change of viral titer is one of the most direct and convincing parameters in anti-virus research. In order to further determine the level of inhibition, PRRSV YN-1 strain was diluted by 1:10 series and added into Marc-145 cells 48 hours post siRNA transfection in 96-well plates. Cytopathic effects (CPE) were monitored until 3 days post virus infection. Viral titers were measured by TCID_50_ assay. We found that compared with the siScramble control group, RACK1 knockdown could significantly protect Marc-145 cells from cytopathic effects (Fig. 5A) and reduced the viral titer by almost 40 fold (Fig. 5B), although non-targeting siRNA knockdown slightly affected the viral titer (3 fold, which is insignificant, with p value > 0.05).

### Knockdown of RACK1 inhibited an ongoing PRRSV infection

**Figure 6:**
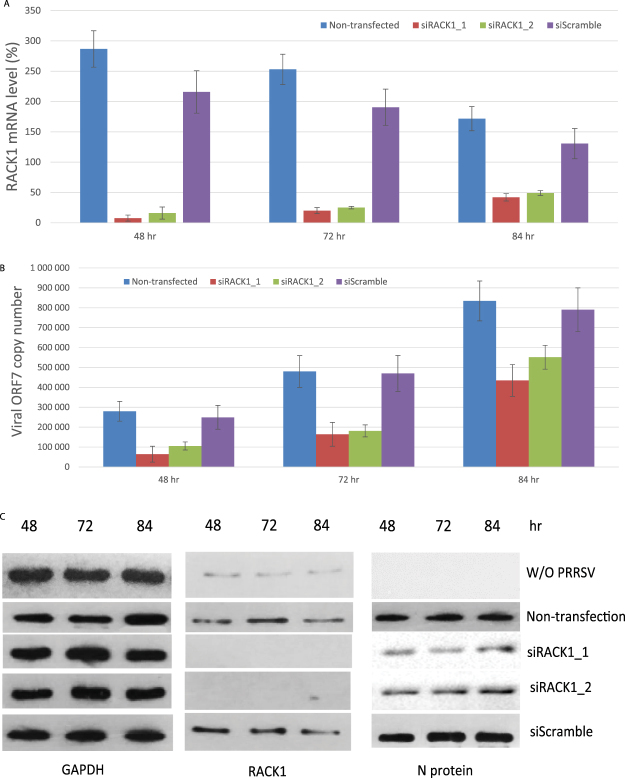
Knockdown of RACK1 inhibited an ongoing PRRSV infection. Marc-145 cells were infected with PRRSV for 12 hours to allow the establishment of the viral infection, followed by siRNA treatments. The mRNA and protein expression level of cellular RACK1 and viral ORF7 were analyzed at different time points after transfection (48, 72 and 84 hours post transfection). (**A**) Cellular RACK1 mRNA expression level. (**B**) Absolute copy number of viral ORF7 mRNA. (**C**) Western blot of cellular RACK1 and viral N protein level. GAPDH was used as internal control in both RT-qPCR and western blot analysis. These data is representative for three independent experiments.

The above described data was obtained from the experiments performed by knocking down RACK1 and then infecting Marc -145 cells with PRRSV. To investigate whether knockdown of RACK1 can inhibit an ongoing PRRSV infection, we first infected Marc-145 cells with 25 TCID50/100ul of PRRSV. Twelve hours post PRRSV infection, the Marc-145 cells were trypsinized for siRNA knockdown. The mRNA and protein expression level of cellular RACK1 and viral ORF7 were analyzed at different time points after transfection (48, 72 and 84 hours post transfection). The data in Fig. 6 clearly showed that compared with the non-targeting siRNA control (siScramble), RACK1 siRNA knockdown significantly reduced the mRNA and protein expression level of cellular RACK1 and viral ORF7, suggesting that knockdown of RACK1 could inhibit an ongoing PRRSV infection.

## Material and Methods

### Virus and Marc-145 cells

A highly pathogenic PRRSV field strain YN-1 (GenBank accession number: KJ747052) was isolated from the lungs of an infected pig in Yunnan province (China) during a severe PRRSV outbreak in 2008, which belongs to the PRRSV genotype 2. Marc-145 cells were used in this study, as PRRSV can replicate in this cell culture system, which has been widely used in the PRRSV research. The Marc-145 cells and PRRSV field strain YN-1 were acquired and applied in our previous study^32^. Marc-145 cells were cultured in Dulbecco’s modified Eagle’s medium (DMEM, Invitrogen) supplemented with 10% heat-inactivated fetal bovine serum (FBS, GIBCO, pH 7.4), 2 mM L-glutamine, 100 U/ml penicillin and 100 μg/ml streptomycin (Invitrogen). The cultures were maintained in a 5% CO_2_ humidified incubator at 37 °C.

### RACK1 mRNA sequence determination from Marc-145 cells

Primer pair for amplification of RACK1 mRNA from Marc-145 cells was designed according to the RACK1 mRNA sequences publically available from The National Center for Biotechnology Information (https://www.ncbi.nlm.nih.gov/). As we previously described^32^, total RNA was isolated from Marc-145 using the RNAiso Plus RNA isolation kit (Takara Dalian, China), and subjected to reverse transcription (EasyScript First-Strand cDNA Synthesis Super Mix, Cat. #AE301, TRANS) and PCR amplification (2× TransTaq High Fidelity (HIFI) PCR SuperMix II, Cat. #AS131, TRANS). The reverse transcription was performed in 10 µl reaction and incubated at 42 °C for 30 mins and then at 85 °C for 5 s, containing 5 µl of 2× ES, 0.5 µl of ES, 1 µl of primer RACK1_R_seq (1 pmol) and 3.5 µl of RNA template. The PCR reaction was carried out in 25 µl reaction, containing 12.5 µl of 2× TransHiFi SuperMix II, 0.5 µl of primer RACK1_F_seq (0.1 pmol), 0.5 µl of primer RACK1_R_seq (0.1 pmol), 1 µl of cDNA template (about 400 ng) and 10.5 µl of water. The amplification program was 94 °C for 5 min and 35 cycles of 94 °C for 30 s, 60 °C for 30 s and 72 °C for 50 s, followed by additional 5 mins extension at 72 °C. The primer pairs used in this study are designed based on the reference sequence XM_011745042 and listed in Table 1.

**Table 1:** Oligonucleotides used in this study.

Name	Sequence 5′—3′)
RACK1_F_seq	CGCCACCATGACTGAGCAGAT
RACK1_R_seq	CTAGCGCGTGCCGATGGT
siRACK1_1 (sense)	ccaucaagcuauggaauacdTdT
siRACK1_1 (antisense)	guauuccauagcuugauggdTdT
siRACK1_2 (sense)	caaauacacuguccaggaudTdT
siRACK1_2 (antisense)	auccuggacaguguauuugdTdT
siScramble (sense)	uucuccgaacgugucacgudTdT
siScramble (antisense)	acgugacacguucggagaadTdT
RACK1_F	TTCTCCTCTGACAACCGGCA
RACK1_R	GCCATCCTTGCCTCCAGAA
Viral N_F	AATGGCCAGCCAGTCAATCA
Viral N_R	TCATGCTGAGGGTGATGCTG
IFNα_F	gtgctcagctgcaagtcaag
IFNα_R	tggtttgagccttttggaac
GAPDH_F	TGGAAAAACCTGCCAAGTACG
GAPDH_R	ATGAGGTCCACCACCCTGTT

CGCCACC, as the conserved sequence of Kozak sequence, is tagged to the 5′ of the start codon of the forward primer sequence to amplify the full sequence of the RACK1 mRNA.

PCR amplicon was electrophoresed using 1.2% agarose gel, purified using the SanPrep column DNA gel purification kit from Sangon (Shanghai, China, Cat. #B518131) and subjected to ligation at 4 °C for overnight. The ligation was carried out in 10 µl reaction, containing 5 µl of Solution I, 50 ng of pMD-18T Vector (pMD-18T Vector cloning kit, Takara, Cat. #3270) and 200–1000 ng cDNA template. The ligation reaction was transformed into competent DH5α cells (Takara, Cat. #9057) which were cultured according to the instruction. Subsequently, the plasmid was extracted using SanPrep Column Plasmid DNA Extraction Kit (Sangon, Cat. #B518191) and confirmed by BamH I (Takara, Cat. #1010A) digestion. Then the RACK1 mRNA was sequenced by Sangon (Shanghai, China), analyzed using Megalign and SeqMan modules under the DNAStar package (version 7.1) and submitted to GenBank (access number: KT751174.1, https://www.ncbi.nlm.nih.gov/nuccore/975883998).

### siRNA transfection and virus infection

Cell seeding, siRNAs transfection and virus infection were performed according to our previous study^32^. In brief, Marc-145 cells were seeded into 96-well plates (10^4^ cells per well) or 6-well plates (3 × 10^5^ cells per well) during reverse transfection with 40 nM siRNA (against RACK1 or with random sequence) and Lipofectamine 3000 (Thermofisher Scientific, Cat. # L3000008) (1.5 µl/well for 96-well plate or 6 µl/well for 6-well plate). Forty-eight hours post transfection, the cells were inoculated with PRRSV YN-1 strain (25 TCID_50_/well, equivalent to an approximate MOI of 0.02,) till further analysis. Each treatment was performed in triplicate. The siRNAs used in this study are listed in Table 1 and purchased from Sangon (Shanghai, China). Transfection with siScramble serves as neutral control for normalization.

### Isolation of total RNA, Reverse transcription and qPCR analysis

As we previously described^32^, total RNA was isolated from Marc-145 cells at different time points post transfection and PRRSV infection using the RNAiso Plus RNA isolation kit (Takara Dalian, China, Cat. #9108/9109), and subjected to reverse transcription (Takara PrimerScript RT reagents kit, Cat. #RR037A) and qPCR analysis (Clontech, SYBR Primer Ex Taq II kit, Cat. # RR820Q). The reverse transcription was performed in 10 µl reaction incubated at 37 °C for 15 mins and then at 85 °C for 5 s, containing 2 µl of 5× Prime Script Buffer, 0.5 µl of Prime Script RT Enzyme Mix 1, 0.5 µl of Oligo dT primer, 0.5 µl of Random 6 mers, 3 µl of RNA (480 ng) and 3.5 µl of water. The qPCR reaction was carried out in 20 µl reaction, containing 10 µl of SYBR Premix Ex Taq^TM^ II (2×), 0.4 µM forward primer, 0.4 µM reverse primer and 1.5 µl of cDNA template (480 ng). The primer pairs used in this study are listed in Table 1. Amplification and detection of samples were performed with the CFX96 Touch Real-Time PCR Detection System (Bio-Rad, USA). The ΔΔCt method^33^ (ABI PRISM 7700 Sequence Detection System. 1999. User Bulletin # 2.) for relative quantification of gene expression was applied to determine viral RNA (ORF7) and cellular RACK1 and IFNα mRNA levels using SYBR Green real-time PCR. GAPDH served as an internal control.

### Western blot analysis

Total viral and cellular proteins were isolated from Marc-145 cells in 6-well plates at different time points as indicated with or without PRRSV infection, according to our previous report^32^. The collected protein samples were resolved under reducing and denaturing conditions using sodium dodecyl sulfate-polyacrylamide gel electrophoresis (SDS-PAGE) and 8% Bis-Tris Novex NuPage gels in conjunction with running buffer. Resolved proteins were transferred to nitrocellulose membranes for western blot detection and blocked at room temperature for 1 h in PBST containing 5% (w/v) dehydrated milk and 0.05% Tween 20 with shaking.

Viral N protein or cellular proteins were probed by overnight incubation at 4 °C with rocking, with the primary antibodies, which was diluted in filtered 5% milk–PBST at a ratio of 1:500 (anti-N protein monoclonal antibody, VMRD, Cat. #080728–004, mouse origin), 1:1000 (anti- GAPDH polyclonal antibody, Proteintech, Cat. #20536-1-AP, rabbit origin), 1:1000 (anti-p65 antibody, Lsbio, Cat. #LS-C352792, rabbit origin), 1:1000 (anti-phospho-p65 Ser536 antibody, Cell Signaling Technology, Cat. #3033 s, rabbit origin), 1:1000 (anti-RACK1 antibody, Cell Signaling Technology, Cat. #5432 s, rabbit origin), or 1:1000 (anti-phospho-IκBα Ser32/36, Cell Signaling Technology, Cat. #9246, mouse origin), followed by secondary goat anti-mouse conjugated horseradish peroxidase (HRP) (Proteintech, Cat. #SA00001-1) or goat anti-rabbit-conjugated horseradish peroxidase (Proteintech, Cat. #SA00001-2) antibody diluted in filtered 5% milk–PBST at a ratio of 1:2,000 and incubated for 1 h at room temperature with rocking. Subsequently, western blots were treated with chemiluminescent ECL Plus substrate (Pierce, Rockford, IL) and imaged using chemiluminescent film (Kodak, Rochester, New York).

### Indirect immunofluorescence staining

According to our previous study^32^, 24 or 48 hours post PRRSV infection, with or without siRNA transfection, the Marc-145 cells were washed with PBS (0.05 M, pH7.4) and fixed with 4% paraformaldehyde (PFA) at room temperature for 10–15 minutes. Then the cells were washed with PBS for three times, permeabilizated with PBS containing 0.3% Triton X-100 for 15 minutes and blocked with PBS containing 1% BSA for 2 hours at 4 °C. The nuclei staining with 5 µg/ml of Hoechst 33342 (Life Technology) was carried out for 20 minutes at room temperature. The cells were subsequently co-incubated with PRRSV antibody against N protein (encoded by ORF7) (VMRD, Cat. #080728-004, mouse origin, 5 µg/ml) and anti-RACK1 antibody (Cell Signaling Technology, Cat. #5432 s, rabbit origin, 2.7 µg/ml) at 4 °C overnight, washed three times with PBS and then co-incubated with Alexa Fluor 488 conjugated goat anti-mouse IgG (H + L) antibody (Proteintech, Cat. #861163) and Alexa Fluor 546 conjugated goat anti-rabbit IgG (H + L) antibody (Thermofisher Scientific, Cat. #11010) at 5 µg/ml for each for 1 h at 37 °C. Staining with Hoechst 33342 (Sigma-Aldrich, Cat. #B2261) at room temperature for 15 minutes was used to visualize the nuclei. After three times PBS wash, the cells were subjected to image analysis by fluorescence microscopy (Olympus).

### Virus titration

As described in our previous study^32^, Marc-145 cells were seeded into 96-well plates (10^4^ cells/well in 100 µl) during siRNA transfection. Forty-eight hours post transfection, a 10× serial dilution of PRRSV YN-1 strain was prepared and each dilution was added into six wells (100 µl/well). CPE was recorded using the inverted microscope over a period of 3 days post virus challenge. Cell number was counted and the 50% tissue culture infected dose (TCID_50_) was determined by Reed–Muench method.

## Discussion

The PRRSV shows a restricted tropism for subsets of porcine macrophages *in vivo*. To date, at least seven cellular molecules have been described as putative receptors for PRRSV, including heparan sulfate, vimentin, CD151, sialoadhesin (CD169), dendritic cell-specific intercellular adhesion melecule-3-grabbing non-integrin (DC-SIGN; CD209), vimentin and CD163^34,35^. Incubation of macrophages at 37 °C with both sialoadhesin- and CD163-specific antibodies completely blocked the PRRSV infection^36^. However, most of the PRRSV receptors were identified on porcine alveolar macrophages (PAMs) with specific functions for PRRSV pathogenesis, for instance, heparan sulphate for binding^37,38^ and sialoadhesin for binding and internalization^39^. Only CD163 was described to be essential in PRRSV infection of non-permissive cells (for example Marc-145 cells, which were used in this study)^36,40^. Therefore, we speculate that additional host factors are needed for PRRSV to complete the viral life cycle in PAMs or Marc-145 cells.

RACK1 is highly conserved across species^41,42^. Therefore, in this study, degenerate polymerase chain reaction (PCR) primers, based on highly conserved regions of RACK1 from the 26 species used to construct the phylogenetic tree, were used in a reverse transcriptase polymerase chain reaction (RT-PCR) reaction to amplify RACK1 from Marc-145 cells. The deduced protein sequence of RACK1 cDNA with a full length of 954 bp from Marc-145 cells shows that it contains 317 amino acid residues, and shares 100% identity with human and porcine (Fig. 1C). Therefore an antibody against human RACK1 could be applied in this study.

A number of pathogenic viruses, such as Epstein–Barr virus^43^ and hepatitis C virus^44^, exploit the NF-κB system for their own profit. PRRSV is recognized as one of the most important viruses for the swine industry, mainly due to its persistence in pigs for quite a long time after initial infection. NF-κB activation has been believed to be one of the key pathogenic mechanisms. Several studies showed that PRRSV infection could trigger NF-κB signal pathway in MARC-145 cells or porcine alveolar macrophages^31,45–47^. The NF-κB activating viral proteins include nucleocapsid (N) protein^47^ and NSP2 protein^48^. However, there are also some controversial data on the interactions between PRRSV and the NF-κB pathway. Some studies reported that the NF-κB deactivating viral proteins NSP2^49^ and NSP1a^45,46,50^ could inhibit the NF-κB signaling pathway by interfering with the poly-ubiquitination process of IκBα. PRRSV may have developed sophisticated strategies to either activate or inhibit NF-κB for its own benefit at different stages of its life cycle. Here our data showed that infection of Marc-145 cells with YN-1 strain which belongs to the PRRSV genotype 2 resulted in phosphorylation of IκBα and p65, 1 hpi and 4 hpi, respectively, signifying NF-κB activation (Fig. 3D,F). Our results are consistent with some previous studies reporting that phosphorylation of IκBα was observed already 30 minutes post infection or 24 hours post transfection in PAMs challenged with PRRSV or in CRL2843 cells transfected with N protein expression vector^31,45–47^. The slight difference may stem from the specific cell type, PRRSV strain and multiplicity of infection. We speculate that sampling from more time points very early in *in vitro* infection (i.e., from 30 mins to 4 hrs) would aid in profiling NF-κB activation.

RACK1 was identified as a novel negative regulator of NF-κB signaling physically associated with the IKK complex in a TNF-triggered manner in 293 T cells^13^. This interaction interferes with the recruitment of the IKK complex to TRAF2, which is a critical step for IKK phosphorylation and subsequent activation triggered by TNF. However, in this study we demonstrated RACK1 as a positive regulator of NF-κB signaling induced by PRRSV infection in Marc-145 cells (Fig. 3). The complete opposite functionality of RACK1 in NF-κB signaling probably stem from the differing stimuli and cell types. The elaborate mechanisms by which PRRSV regulates NF-κB activation and how RACK1 plays its roles in NF-κB and PRRSV replication require further studies.

There was a report showing that viral replication of PRRSV strain CH-1a was not obviously affected when bone marrow-derived macrophages (BMDMs) were treated with of NF-κB inhibitor BAY11-7082^51^. Hence we reasoned that inhibition of NF-κB signaling may not interfere with the replication of PRRSV strain YN-1 in Marc-145 cells, and that the inhibition of YN-1 replication (Fig. 4) and reduction of viral titer (Fig. 5) by RACK1 siRNA knockdown was not through deactivation of NF-κB. This also needs further investigation.

Fighting viral infections is hampered by the scarcity of viral targets and their variability. Viruses depend on cellular molecules, which are attractive alternative targets for drug development, provided that they are dispensable for normal cell function. Inhibition of RACK1 does not affect *Drosophila* or human cell viability and proliferation, and RACK1-silenced adult flies are viable, indicating that this protein is not essential for general translation^14^. A similar phenotype was observed in this study, as shown in Figs 3, 4 and 5 in terms of cell number.

Collectively, in spite of the controversy about whether PRRSV infection activates or deactivates the NF-κB pathway, our results demonstrate that the highly pathogenic PRRSV strain YN-1 isolated by our lab did activate the NF-κB signal pathway and upregulate RACK1 expression over the investigation time period in Marc-145 cells. The authors suggested that RACK1 is very conservative across different species based on the phylogenetic analysis of mRNA and deduced amino acid sequences. For the first time we demonstrated that siRNA knockdown of RACK1 inhibited PRRSV replication in Marc-145 cells, abrogated the NF-κB activation induced by PRRSV infection and eventually reduced the viral titer. Furthermore, we demonstrated that knockdown of RACK1 could inhibit an ongoing PRRSV infection. The conclusion can be drawn that RACK1 is an indispensable cellular factor for PRRSV replication in Marc-145 cells and thus NF-κB activation, while dispensable for cell viability. This study may provide some insights into the molecular mechanisms of PRRSV infection in swine and RACK1 was uncovered as a promising potential therapeutic target for PRRSV intervention. The therapeutic potential of RACK1 could be realised by small molecules which can selectively target RACK1or/and its interacting proteins^52^. A small molecule screen is currently carried out in our lab to identify chemical compounds for inhibition of PRRSV replication by downregulating the RACK1 expression level.

### Availability of data and materials

The datasets supporting the findings can be found in the tables and figures of the manuscript.

## Electronic supplementary material

Supplementary figures

## References

[1] Pejsak Z, Markowska-Daniel I (1997). Losses due to porcine reproductive and respiratory syndrome in a large swine farm. Comp Immunol Microbiol Infect Dis..

[2] Neumann EJ (2005). Assessment of the economic impact of porcine reproductive and respiratory syndrome on swine production in the United States. J. Am. Vet. Med. Assoc..

[3] Meulenberg JJ (2000). PRRSV, the virus. Vet. Res..

[4] Snijder EJ, Meulenberg JJ (1998). The molecular biology of arteriviruses. J. Gen. Virol..

[5] Wang FX (2012). Role of non-structural protein 2 in the regulation of the replication of the porcine reproductive and respiratory syndrome virus in MARC-145 cells: effect of gene silencing and over expression. Vet. Microbiol..

[6] Guo DW (2013). Identification of the interaction between porcine reproductive and respiratory syndrome virus NSP9 protein and porcine RACK1. Chinese journal of preventive veterinary medicine.

[7] Ron D, Mochly-Rosen D (1994). Agonists and antagonists of protein kinase C function, derived from its binding proteins. J Biol Chem..

[8] Fei L (2017). RACK1 promotes lung cancer cell growth via an MCM7/RACK1/ Akt signaling complex. Oncotarget..

[9] Shi S (2012). RACK1 promotes non-small-cell lung cancer tumorigenicity through activating sonic hedgehog signaling pathway. J Biol Chem..

[10] Deng YZ (2012). RACK1 suppresses gastric tumorigenesis by stabilizing the β-catenin destruction complex. Gastroenterology..

[11] Yao F (2014). RACK1 modulates NF-κB activation by interfering with the interaction between TRAF2 and the IKK complex. Cell Res..

[12] Li JJ, Xie D (2015). RACK1, a versatile hub in cancer. Oncogene..

[13] Duff D, Long A (2017). Roles for RACK1 in cancer cell migration and invasion. Cell Signal..

[14] Majzoub K (2014). RACK1 controls IRES-mediated translation of viruses. Cell..

[15] Demirov D, Gabriel G, Schneider C, Hohenberg H, Ludwig S (2012). Interaction of influenza A virus matrix protein with RACK1 is required for virus release. Cell Microbiol..

[16] Gallina A, Rossi F, Milanesi G (2001). Rack1 binds HIV-1 Nef and can act as a Nef-protein kinase C adaptor. Virology..

[17] Jia B (2015). Hepatitis B virus core protein sensitizes hepatocytes to tumor necrosis factor-induced apoptosis by suppression of the phosphorylation of mitogen-activated protein kinase kinase 7. J Virol..

[18] Tonganunt M, Saelee N, Chotigeat W, Phongdara A (2009). Identification of a receptor for activated protein kinase C1 (Pm-RACK1), a cellular gene product from black tiger shrimp (Penaeus monodon) interacts with a protein, VP9 from the white spot syndrome virus. Fish Shellfish Immunol..

[19] Daniels CC, Rovnak J, Quackenbush SL (2008). Walleye dermal sarcoma virus Orf B functions through receptor for activated C kinase (RACK1) and protein kinase C. Virology..

[20] Kubota T, Yokosawa N, Yokota S, Fujii N (2002). Association of mumps virus V protein with RACK1 results in dissociation of STAT-1 from the alpha interferon receptor complex. J Virol..

[21] Lin W (2015). The association of receptor of activated protein kinase C 1(RACK1) with infectious bursal disease virus viral protein VP5 and voltage-dependent anion channel 2 (VDAC2) inhibits apoptosis and enhances viral replication. J Biol Chem..

[22] Zhang Y (2017). Integrin beta and receptor for activated protein kinase C are involved in the cell entry of Bombyx mori cypovirus. Appl Microbiol Biotechnol..

[23] Zhang CC (2014). Screening of cellular proteins that interact with the classical swine fever virus non-structural protein 5A by yeast two-hybrid analysis. Journal of Biosciences..

[24] Ling LJ (2012). Identification of the interaction between classical swine fever virus E2 protein and porcine RACK1. Chinese Journal of Preventive Veterinary Medicine..

[25] Tardif M, Savard M, Flamand L, Gosselin J (2002). Impaired protein kinase C activation/translocation in Epstein-Barr virus-infected monocytes. J Biol Chem..

[26] Choi DS, Young H, McMahon T, Wang D, Messing RO (2003). The mouse RACK1 gene is regulated by nuclear factor-kappa B and contributes to cell survival. Mol Pharmacol..

[27] Jia D (2013). Up-regulation of RACK1 by TGF-β1 promotes hepatic fibrosis in mice. PLoS One..

[28] Karin M, Cao Y, Greten FR, Li ZW (2002). NF-kappaB in cancer: from innocent bystander to major culprit. Nat Rev Cancer..

[29] Li Q, Verma IM (2002). NF-kappaB regulation in the immune system. Nat Rev Immunol..

[30] Hayden MS, Ghosh S (2004). Signaling to NF-kappaB. Genes Dev..

[31] Fu Y (2012). Porcine reproductive and respiratory syndrome virus induces interleukin-15 through the NF-κB signaling pathway. J Virol..

[32] Zheng L (2015). Inhibition of porcine reproductive and respiratory syndrome virus replication *in vitro* using DNA-based short antisense oligonucleotides. BMC Vet Res..

[33] Pfaffl MW (2001). A new mathematical model for relative quantification in real time RT-PCR. Nucleic Acids Research..

[34] Zhang Q, Yoo D (2015). PRRS virus receptors and their role for pathogenesis. Vet Microbiol..

[35] Shi C, Liu Y, Ding Y, Zhang Y, Zhang J (2015). PRRSV receptors and their roles in virus infection. Arch Microbiol..

[36] Van Gorp H, Van Breedam W, Delputte PL, Nauwynck HJ (2008). Sialoadhesin and CD163 join forces during entry of the porcine reproductive and respiratory syndrome virus. J Gen Virol..

[37] Vanderheijden N, Delputte P, Nauwynck H, Pensaert M (2001). Effects of heparin on the entry of porcine reproductive and respiratory syndrome virus into alveolar macrophages. Adv Exp Med Biol..

[38] Delputte PL, Vanderheijden N, Nauwync HJ, Pensaert MB (2002). Involvement of the matrix protein in attachment of porcine reproductive and respiratory syndrome virus to a heparinlike receptor on porcine alveolar macrophages. J Virol..

[39] Vanderheijden N (2003). Involvement of sialoadhesin in entry of porcine reproductive and respiratory syndrome virus into porcine alveolar macrophages. J Virol..

[40] Calvert JG (2007). CD163 expression confers susceptibility to porcine reproductive and respiratory syndrome viruses. J Virol..

[41] Kwak JM (1997). Insulin-induced maturation of Xenopus oocytes is inhibited by microinjection of a Brassica napus cDNA clone with high similarity to a mammalian receptor for activated protein kinase C. Planta..

[42] Chou YC (1999). Structure and genomic organization of porcine RACK1 gene. Biochim Biophys Acta..

[43] Mosialos G (1995). The Epstein-Barr virus transforming protein LMP1 engages signaling proteins for the tumor necrosis factor receptor family. Cell..

[44] You LR, Chen CM, Lee YH (1999). Hepatitis C virus core protein enhances NF-kappaB signal pathway triggering by lymphotoxin-beta receptor ligand and tumor necrosis factor alpha. J Virol..

[45] Lee SM, Kleiboeker SB (2005). Porcine arterivirus activates the NF-kappaB pathway through IkappaB degradation. Virology..

[46] Luo R (2008). Porcine reproductive and respiratory syndrome virus (PRRSV) suppresses interferon-beta production by interfering with the RIG-I signaling pathway. Mol Immunol..

[47] Luo R (2001). Activation of NF-κB by nucleocapsid protein of the porcine reproductive and respiratory syndrome virus. Virus Genes..

[48] Fang Y (2012). Porcine reproductive and respiratory syndrome virus nonstructural protein 2 contributes to NF-κB activation. Virol. J..

[49] Sun Z, Chen Z, Lawson SR, Fang Y (2010). The cysteine protease domain of porcine reproductive and respiratory syndrome virus nonstructural protein 2 possesses deubiquitinating and interferon antagonism functions. J. Virol..

[50] Song C, Krell P, Yoo D (2010). Nonstructural protein 1α subunit-based inhibition of NF-κB activation and suppression of interferon-β production by porcine reproductive and respiratory syndrome virus. Virology..

[51] Hou J (2012). Induction of interleukin-10 is dependent on p38 mitogen-activated protein kinase pathway in macrophages infected with porcine reproductive and respiratory syndrome virus. Virol. J..

[52] Ron D (2013). RACK(1) to the future – a historical perspective. Cell Commun Signal..

