# Supplementary material for: RACK1 is indispensable for porcine reproductive and respiratory syndrome virus replication and NF-κB activation in Marc-145 cells
Source: Sci Rep. 2018 Feb 14;8:2985. doi: 10.1038/s41598-018-21460-4 (PMC5813008; doi:10.1038/s41598-018-21460-4)
Supplement: Supplementary file 1 — Supplementary figures [file 41598_2018_21460_MOESM1_ESM.pdf]

RACK1 is indispensable for porcine reproductive and respiratory syndrome virus replication and NF-κB activation in Marc-145 cells

Junlong Bi[1, 2, 3, §], Qian Zhao[2, §], Lingyun Zhu[2, 4], Xidan Li[5], Guishu Yang[2], Jianping Liu[5, *], Gefen Yin[2, *]

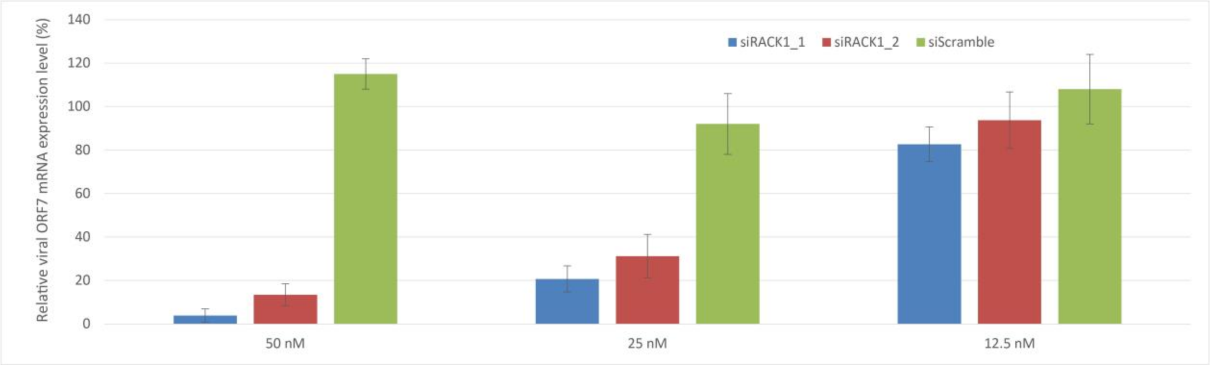

Supplementary Figure 1. Inhibition of PRRSV replication by knockdown of RACK1 in Marc-145 cells is dose dependent. Forty-eight hours post siRNA knockdown with three different concentrations of siRNAs (50 nM, 25 nM and 12.5 nM), Marc-145 cells were inoculated with PRRSV YN-1 strain (25 TCID50). Viral ORF7 mRNA expression level was measured by RT-qPCR 60 hours post siRNA knockdown. The histograms shown here are representative data from three independent experiments.

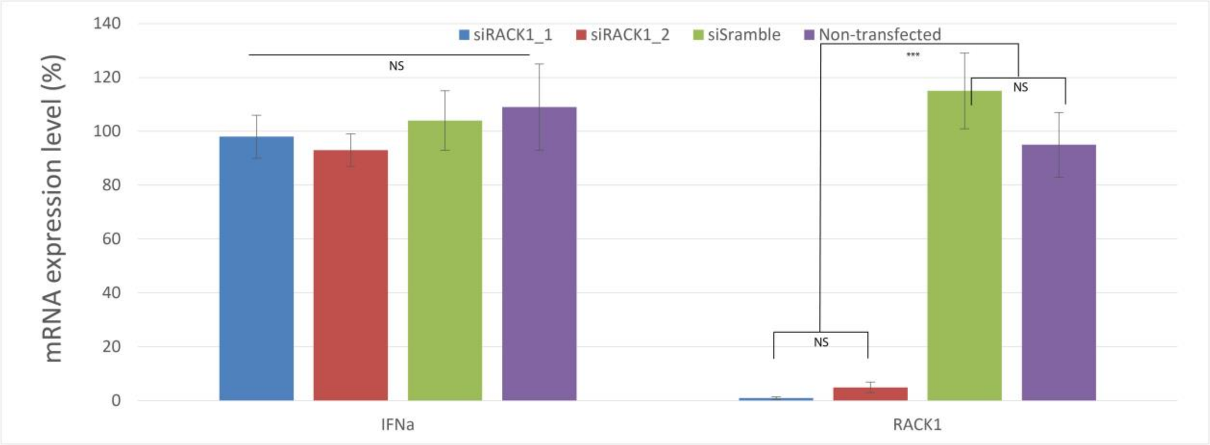

Supplementary Figure 2. RACK1 siRNA knockdown in Marc-145 cells did not significantly change the IFNa mRNA level. Relative mRNA expression level of IFNα and RACK1 was analyzed 48 hours post siRNA transfection. The ΔΔCt method for relative quantification of gene expression was used and GAPDH was used as internal control. NS: not significant. ***: p value <0.005. The histograms and blots shown here are representative data from three independent experiments.
